# Supplementary material for: A thalamocortical pathway for fast rerouting of tactile information to occipital cortex in congenital blindness
Source: Nat Commun. 2019 Nov 14;10:5154. doi: 10.1038/s41467-019-13173-7 (PMC6856176; doi:10.1038/s41467-019-13173-7)
Supplement: Supplementary file 1 — Supplementary Information [file 41467_2019_13173_MOESM1_ESM.pdf]

Supplementary Information

A thalamocortical pathway for fast rerouting of tactile information to occipital cortex in congenital blindness

Franziska Müller[1§], Guiomar Niso[2,3,4§], Soheila Samiee[2], Maurice Ptito[1,5], Sylvain Baillet[2*], Ron Kupers[1,5,67*]

1. BRAINlab, Department of Neuroscience, Panum Institute, University of Copenhagen, Denmark
2. McConnell Brain Imaging Centre, Montreal Neurological Institute, McGill University, Montreal, Qc, Canada
3. Centre for Biomedical Technology, Universidad Politécnica de Madrid, Madrid, Spain
4. Biomedical Image Technologies, ETSI Telecomunicación, Universidad Politécnica de Madrid and CIBER-BBN, Spain
5. École d'Optométrie, Canada, Université de Montréal, Montréal, Qc, Canada
6. Department of Radiology & Biomedical Imaging, Yale University, New Haven, CT, USA
7. Institute of Neuroscience, Université catholique de Louvain, Brussels, Belgium

[§] These authors contributed equally to this work

Supplementary Table 1: Demographic characteristics blind participants.

Participant	Cause of blindness	age	sex	handedness	Braille Reading *hours/day*	Braille Reading *wpm*
CB1	ROP	26	M	R	1h	55
CB2	ROP	28	F	R	0.5 h	85
CB3	ROP	29	M	R	0.5 h	80
CB4	ROP	32	F	R	0.5 h	187
CB5	Retinoblastoma	48	M	R	1.5 h	158
CB6	ROP	53	F	R	2 h	100
CB7	ROP	53	M	L	1h	130
CB8	ROP	62	M	R	3.5 h	147

Abbreviations: wpm = words per minute

Supplementary Table 2: The eight conditions of a normal trial.

		Stimulation			
		Left index finger		Right index finger	
		SUPRA	CL	SUPRA	CL
Attention	Left	L SUPRA1	L CL1	R SUPRA0	R CL0
	Right	L SUPRA0	L CL0	R SUPRA1	R CL1

The eight different conditions are the result of the 2 x 2 x 2 design, using two stimulation sides (Left finger stimulation = L, Right finger stimulation = R), two stimulation intensities (Supra-threshold intensity = SUP; Comfort Limit = CL) and two attentional conditions (attended = 1, unattended = 0).

Supplementary Table 3: Amount of trials of the different trial types and conditions.

Normal trials	640 (10 x 64)
L CL1	120 (10 x 12)
L CL0	40 (10 x 4)
L SUPRA1	120 (10 x 12)
L SUPRA0	40 (10 x 4)
R CL1	120 (10 x 12)
R CL0	40 (10 x 4)
R SUPRA1	120 (10 x 12)
R SUPRA0	40 (10 x 4)
Trial-cue only trials	40 (10 x 4)
Non-trial-cue only	40 (10 x 4)
**Total amount of trials**	**720 (10 x 72)**

A total of 720 trials was distributed over 10 sessions, each session consisting of 72 trials. The total of 720 trials is the sum of the different trial types (Normal trials = 640, Trial-cue only trials = 40, Non-trial-cue only trials =40).

Supplementary Table 4: Within and between subject effect for beta coefficients of linear regression model of V1 contributions to MEG data in the 35 – 50 ms interval after stimulus.

Within Subjects Effects

	Sum of Squares	df	Mean Square	F	P
Hemisphere	0.147	1	0.1472	1.70	0.214
Hemisphere ✻ Group	0.202	1	0.2021	2.33	0.149
Residual	1.214	14	0.0867		

Note. Type 3 Sums of Squares

Between Subjects Effects

	Sum of Squares	df	Mean Square	F	p
Group	0.492	1	0.4919	15.6	0.001
Residual	0.441	14	0.0315		

Note. Type 3 Sums of Squares

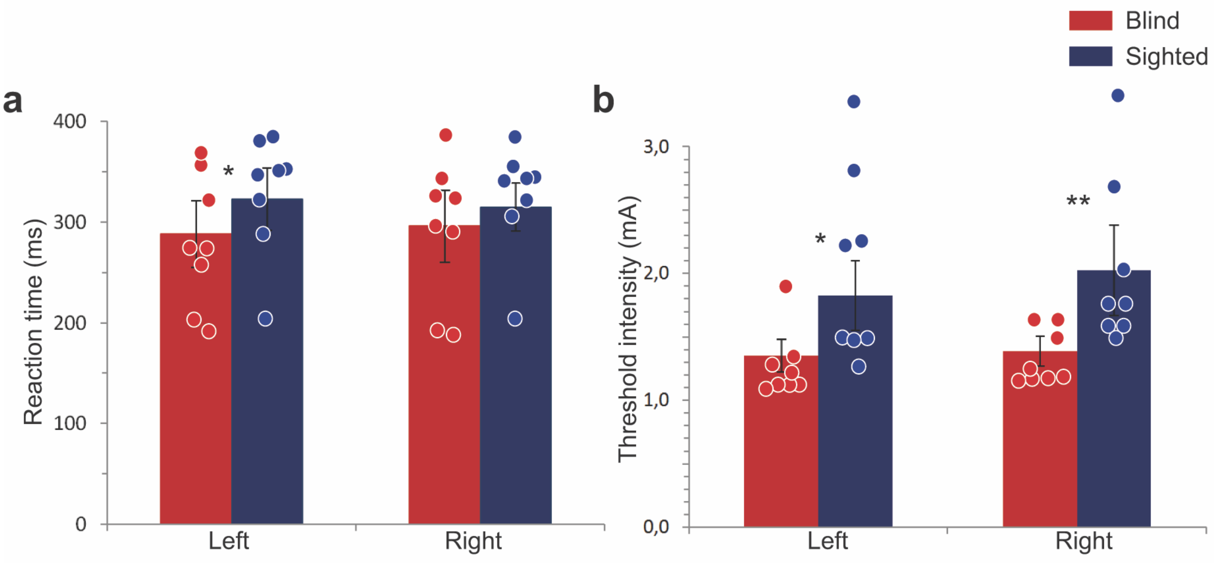

Supplementary Figure 1: Behavioral data. (a) Congenitally blind subjects had faster reaction times to left hand suprathreshold stimulation. The difference was not significant for right hand stimulation. (b) Congenitally blind subjects had significantly lower detection thresholds for both left and right hand stimulation. * = P<0.05; ** = P<0.01 (unpaired student t-test).

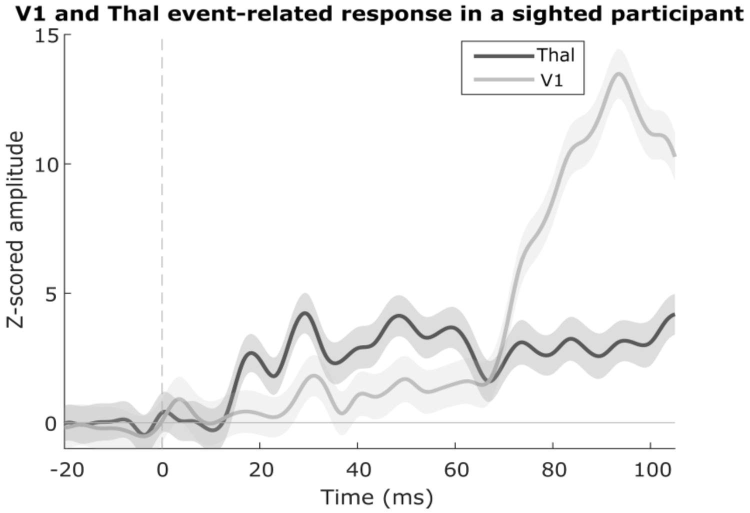

Supplementary Figure 2: Evidence of early thalamic responses in a control visual task, in a sighted healthy subject. The same distributed source modelling procedure was applied on the data from one participant available from a separate publicly available set of MEG data (1). The selected data consisted of the visual presentation in 96 trials of faces to a participant seated under a similar Elekta MEG instrument as the one used to collect our own data. We defined the same ROIs as for the SC and CB groups. The resulting traces show an early response from the posterior aspect of the thalamus following visual presentation (0 ms), which preceded the response peak in primary visual cortex (V1). Shading indicates the standard error on the mean.

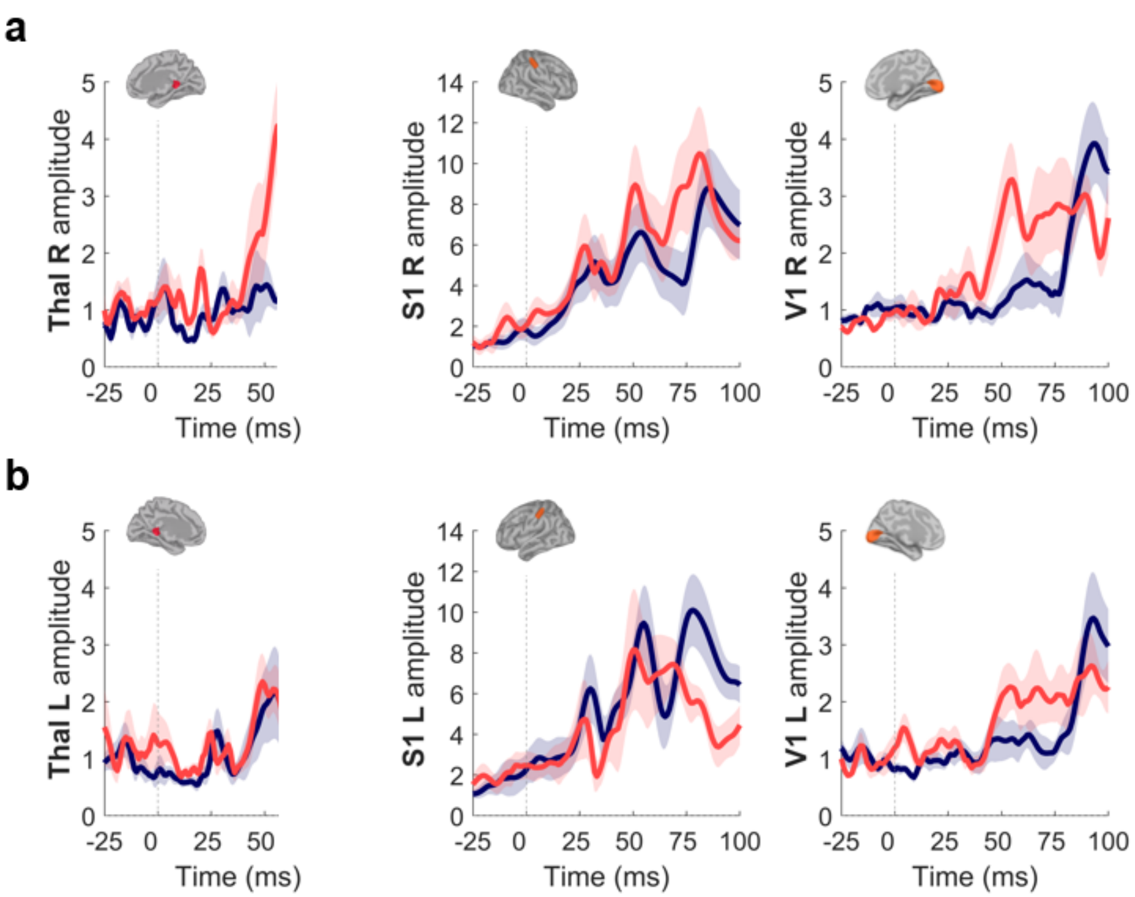

Supplementary Figure 3: Event-related response for left and right index finger stimulation. ROI activations from the full distributed cortical source model following electro-cutaneous stimulation (0ms) of the left (a) and right (b) index finger in contralateral thalamus (Thal), S1 and V1 for congenitally blind (CB, in red) and sighted control (SC, in blue) participants. Shading indicates standard error on the mean estimates.

Supplementary References

1. Wakeman, D.G. & Henson, R.N. A Multi-Subject, Multi-Modal Human Neuroimaging Dataset. Scientific Data 2 (January): 150001. https://doi.org/10.1038/sdata.2015.1 (2015).
