# Supplementary material for: A thalamocortical pathway for fast rerouting of tactile information to occipital cortex in congenital blindness
Source: Nat Commun. 2019 Nov 14;10:5154. doi: 10.1038/s41467-019-13173-7 (PMC6856176; doi:10.1038/s41467-019-13173-7)
Supplement: Supplementary file 3 — Reporting Summary [file 41467_2019_13173_MOESM3_ESM.pdf]

nature research

Corresponding author(s): Ron Kupers

Last updated by author(s): Oct 8, 2019

Reporting Summary

Nature Research wishes to improve the reproducibility of the work that we publish. This form provides structure for consistency and transparency in reporting. For further information on Nature Research policies, see Authors & Referees and the Editorial Policy Checklist.

Statistics

For all statistical analyses, confirm that the following items are present in the figure legend, table legend, main text, or Methods section.

n/a	Confirmed	
☐	☒	The exact sample size (*n*) for each experimental group/condition, given as a discrete number and unit of measurement
☐	☐	A statement on whether measurements were taken from distinct samples or whether the same sample was measured repeatedly
☐	☒	The statistical test(s) used AND whether they are one- or two-sided<br>*Only common tests should be described solely by name; describe more complex techniques in the Methods section.*
☐	☐	A description of all covariates tested
☐	☒	A description of any assumptions or corrections, such as tests of normality and adjustment for multiple comparisons
☐	☐	A full description of the statistical parameters including central tendency (e.g. means) or other basic estimates (e.g. regression coefficient) AND variation (e.g. standard deviation) or associated estimates of uncertainty (e.g. confidence intervals)
☐	☐	For null hypothesis testing, the test statistic (e.g. *F*, *t*, *r*) with confidence intervals, effect sizes, degrees of freedom and *P* value noted<br>*Give P values as exact values whenever suitable.*
☐	☐	For Bayesian analysis, information on the choice of priors and Markov chain Monte Carlo settings
☐	☐	For hierarchical and complex designs, identification of the appropriate level for tests and full reporting of outcomes
☐	☐	Estimates of effect sizes (e.g. Cohen's *d*, Pearson's *r*), indicating how they were calculated

Our web collection on statistics for biologists contains articles on many of the points above.

Software and code

Policy information about availability of computer code

Data collection	*Provide a description of all commercial, open source and custom code used to collect the data in this study, specifying the version used OR state that no software was used.*
Data analysis	*Provide a description of all commercial, open source and custom code used to analyse the data in this study, specifying the version used OR state that no software was used.*

For manuscripts utilizing custom algorithms or software that are central to the research but not yet described in published literature, software must be made available to editors/reviewers. We strongly encourage code deposition in a community repository (e.g. GitHub). See the Nature Research guidelines for submitting code & software for further information.

Data

Policy information about availability of data

All manuscripts must include a data availability statement. This statement should provide the following information, where applicable:

- Accession codes, unique identifiers, or web links for publicly available datasets
- A list of figures that have associated raw data
- A description of any restrictions on data availability

All relevant data is available from the corresponding authors upon reasonable request.

Field-specific reporting

Please select the one below that is the best fit for your research. If you are not sure, read the appropriate sections before making your selection.

☒ Life sciences ☐ Behavioural & social sciences ☐ Ecological, evolutionary & environmental sciences

For a reference copy of the document with all sections, see nature.com/documents/nr-reporting-summary-flat.pdf

Life sciences study design

All studies must disclose on these points even when the disclosure is negative.

Sample size	Our sample size consisted of 8 congenitally blind (CB) subjects, each matched for sex and age to a sighted control participant. Congenitally blind subjects without any residual vision are extremely rare. On top of that, additional requirements (no metal implants, no medication, no neurological or psychiatric antecedents, being able to sit still for 3 hours in a MEG scanner,...) made it impossible to have a larger study population. Our sample size is quite comparable that that of many other studies using functional brain imaging of CB individuals, originating from Western countries where congenital blindness is an extremely rare condition.
Data exclusions	No data were excluded
Replication	We have analyzed our data using two independent statistical approaches. The results of the two analyses were very similar.
Randomization	We used a parallel group design. In order to match our CB subjects with normal sighted controls, we matched each blind participants to a sighted control of the same sex, same age (max 2 years difference) and education level. All blind and sighted participants had full-time professional activities
Blinding	Blinding was not possible as congenitally blind participants are readily distinguishable from sighted controls. This is not a problem for the data collection since we are measuring brain activity in response to electrotactile stimulation of the index finger which is very unlikely to be affected by knowledge of group assignment.

Reporting for specific materials, systems and methods

We require information from authors about some types of materials, experimental systems and methods used in many studies. Here, indicate whether each material, system or method listed is relevant to your study. If you are not sure if a list item applies to your research, read the appropriate section before selecting a response.

Materials & experimental systems

n/a	Involved in the study	
☒	☐	Antibodies
☒	☐	Eukaryotic cell lines
☒	☐	Palaeontology
☒	☐	Animals and other organisms
☐	☒	Human research participants
☒	☐	Clinical data

Methods

n/a	Involved in the study	
☒	☐	ChIP-seq
☒	☐	Flow cytometry
☐	☒	MRI-based neuroimaging

Human research participants

Policy information about studies involving human research participants

Population characteristics	*Describe the covariate-relevant population characteristics of the human research participants (e.g. age, gender, genotypic information, past and current diagnosis and treatment categories). If you filled out the behavioural & social sciences study design questions and have nothing to add here, write "See above."*
Recruitment	*Describe how participants were recruited. Outline any potential self-selection bias or other biases that may be present and how these are likely to impact results.*
Ethics oversight	*Identify the organization(s) that approved the study protocol.*

Note that full information on the approval of the study protocol must also be provided in the manuscript.

Magnetic resonance imaging

Experimental design

Design type	Structural brain scanning (T1-based images) for cor-registration of the MEG data
Design specifications	One anatomical scan of the brain after the MEG experiment
Behavioral performance measures	no behavioral measures

Acquisition

Imaging type(s)	structural
Field strength	3 T
Sequence & imaging parameters	gradient echo (MPRAGE) with the following specifications: (TR = 2420 ms, TE = 3.7 ms, flip angle = 9°, inversion time = 960 ms)
Area of acquisition	whole brain

Diffusion MRI ☐ Used ☒ Not used

Preprocessing

Preprocessing software	none
Normalization	in-house, provided in BRAINSTROM software package
Normalization template	in-house, provided in BRAINSTROM software package
Noise and artifact removal	none
Volume censoring	none

Statistical modeling & inference

Model type and settings	images only used for purposes of MEG-data co-registration
Effect(s) tested	none

Specify type of analysis: ☒ Whole brain ☐ ROI-based ☐ Both

Statistic type for inference (See Eklund et al. 2016)	No statistics. images only used for purposes of MEG-data co-registration
Correction	No correction. images only used for purposes of MEG-data co-registration

Models & analysis

n/a	Involved in the study
☒	☐ Functional and/or effective connectivity
☒	☐ Graph analysis
☒	☐ Multivariate modeling or predictive analysis
